# The genome sequence of the harvestman spider, *Odiellus spinosus *(Bosc, 1792)

**DOI:** 10.12688/wellcomeopenres.22760.1

**Published:** 2024-07-26

**Authors:** Sergio Henriques, Natural History Museum Genome Acquisition Lab, Darwin Tree of Life Barcoding collective, Wellcome Sanger Institute Tree of Life Management, Samples and Laboratory team, Wellcome Sanger Institute Scientific Operations: Sequencing Operations, Wellcome Sanger Institute Tree of Life Core Informatics team, Tree of Life Core Informatics collective, Darwin Tree of Life Consortium

**Affiliations:** 1 Department of Biological Sciences,, Butler University Department of Biological Sciences, Indianapolis, Indiana, USA; 2 Indianapolis Zoological Gardens, Indianapoli, Indiana, USA

**Keywords:** Odiellus spinosus, harvestman spider, genome sequence, chromosomal, Opiliones

## Abstract

We present a genome assembly from an individual female *Odiellus spinosus* (harvestman spider; Arthropoda; Arachnida; Opiliones; Phalangiidae). The genome sequence spans 443.70 megabases. Most of the assembly is scaffolded into 16 chromosomal pseudomolecules. The mitochondrial genome has also been assembled and is 16.07 kilobases in length.

## Species taxonomy

Eukaryota; Opisthokonta; Metazoa; Eumetazoa; Bilateria; Protostomia; Ecdysozoa; Panarthropoda; Arthropoda; Chelicerata; Arachnida; Opiliones; Phalangida; Palpatores; Phalangioidea; Phalangiidae; Oligolophinae; *Odiellus*; *Odiellus spinosus* (Bosc, 1792) (NCBI:txid2803876).

## Background

*Odiellus spinosus* (Bosc, 1792), is an arachnid of the Opiliones order, placed in the family Phalangiidae, a group commonly referred overall as harvestmen, or shepherd spiders in old texts ( Bristowe, 1949). It is the only *Odiellus* species occurring in Britain, where it is widely considered to be an established introduction ( Davidson, 2019) with initial records dating back to the nineteenth century ( Pickard-Cambridge, 1890). The species appears to be expanding northwards ever since. *Odiellus spinosus* is native to southern Europe beyond the United Kingdom it has also been introduced and is rapidly expanding in recent decades, including Belgium ( Van de Poel *et al.*, 2021), Denmark ( Toft, 2018), Germany, Netherlands ( Spoek, 1963; Wijnhoven, 2009), Poland ( Rozwałka *et al.*, 2013), Sweden ( Shah & Coulson, 2021) and Switzerland ( Spoek, 1963).

This is a xerothermophilic species and outside its native range, the species seems to prefer dry open areas, such as sand-dunes ( Spoek, 1963). In the United Kingdom it can be often found in synanthropic biotopes, including gardens, parks, nearby buildings ( Hillyard & Sankey, 1989) and railway embankments ( Bennett, 1962).

This is potentially the largest species of Opiliones in the UK (body length: 6–11 mm), with a distinctively flat body and shorter legs than most members of the group. Identification is further aided by the presence of a prominent trident located in front of its eyes, composed of three large, broad-based spines ( Hillyard & Sankey, 1989; Spoek, 1963). Like other members of the group, males have a long penis for direct insemination (4 mm in *Odiellus spinosus*), and females of this species have very long ovipositors, up to 1.45 times the length of their own bodies, although that is shorter than the impressively long ovipositor of *Phalangium opilio*, which can be up to 2.1 times longer than the female’s body ( Bennett, 1962). Females in captivity have often been reported to lay two batches of eggs (with an interval between batches ranging from 16 to 27 days), laying over 180 eggs each, but able to lay up to 318, with a mean of 245 eggs ( Bennett, 1962). These eggs are large (up to 1.4 mm in diameter) and have not been recorded buried underground (although this might be due to sampling bias) but are rather often found laid beneath decaying wood, leaf litter, rocks (including synanthropic rubble) or under discarded garden vegetation and compost heaps (pers. observation and Bennett, 1962).

In the UK, *Odiellus spinosus* egg laying has been reported as starting in early October ( Bennett, 1962), although in recent years, it has been observed to be taking place later in the year in the London area, potentially due to changing temperatures under climate change. This species overwinters as a non-diapause egg, with the first young hatching in late January (captivity) or early February (in nature), while some were recorded hatching at the end of March ( Bennett, 1962), accounting for a long hatching period of several months. This small detail of the species biology might appear trivial at first, but it could be important to explain the species success in colonizing new areas, as their eggs can clearly survive long journeys, even under cold storage conditions.

The *O. spinosus* life cycle takes approximately one year to complete, with juvenile stages developing over six months (from February to July) and the first specimens moulting into adulthood around the end of July, accounting for a three-month-long adult stage (from August to October), the egg laying season is approximately 6 weeks and before the year ends in December, all adults have perished ( Bennett, 1962).

The genome of *Odiellus spinosus* was sequenced as part of the Darwin Tree of Life Project, a collaborative effort to sequence all named eukaryotic species in the Atlantic Archipelago of Britain and Ireland.

## Genome sequence report

**Figure 1.**
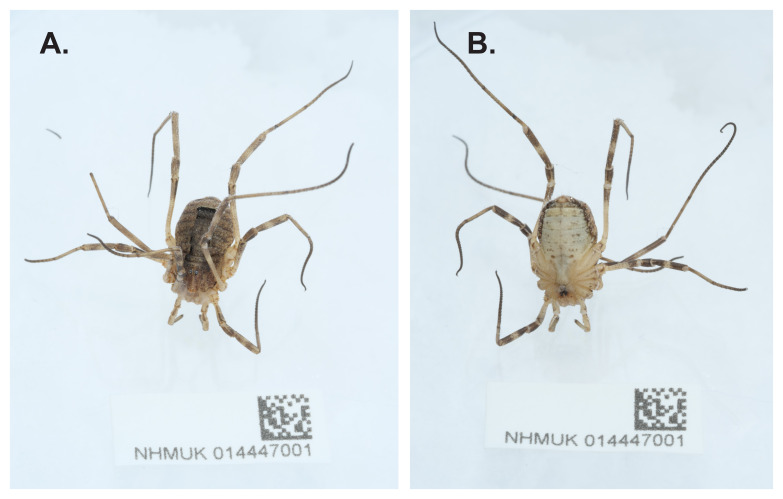
Photograph of the *Odiellus spinosus* (qqOdiSpin1) specimen used for genome sequencing.

The genome of an adult specimen of *Odiellus spinosus* ( Figure 1) was sequenced using Pacific Biosciences single-molecule HiFi long reads, generating a total of 2.23 Gb (gigabases) from 0.24 million reads, providing approximately 54-fold coverage. Primary assembly contigs were scaffolded with chromosome conformation Hi-C data, which produced 143.21 Gbp from 948.41 million reads, yielding an approximate coverage of 323-fold. Specimen and sequencing information is summarised in Table 1.

**Table 1.** Specimen and sequencing data for *Odiellus spinosus*.

Project information			
**Study title**	Odiellus spinosus		
**Umbrella BioProject**	PRJEB59133		
**Species**	*Odiellus spinosus*		
**BioSample**	SAMEA9066027		
**NCBI taxonomy ID**	2803876		
Specimen information			
**Technology**	**ToLID**	**BioSample accession**	**Organism part**
**PacBio long read sequencing**	qqOdiSpin1	SAMEA9066099	cephalothorax
**Hi-C sequencing**	qqOdiSpin2	SAMEA9066097	cephalothorax
**RNA sequencing**	qqOdiSpin3	SAMEA9066096	abdomen
Sequencing information			
**Platform**	**Run accession**	**Read count**	**Base count (Gb)**
**Hi-C Illumina NovaSeq 6000**	ERR10802466	9.48e+08	143.21
**PacBio Sequel IIe**	ERR10809388	2.11e+06	23.96
**PacBio Sequel IIe**	ERR10809389	2.42e+05	2.23
**RNA Illumina NovaSeq 6000**	ERR10890709	6.16e+07	9.3

**Figure 2.**
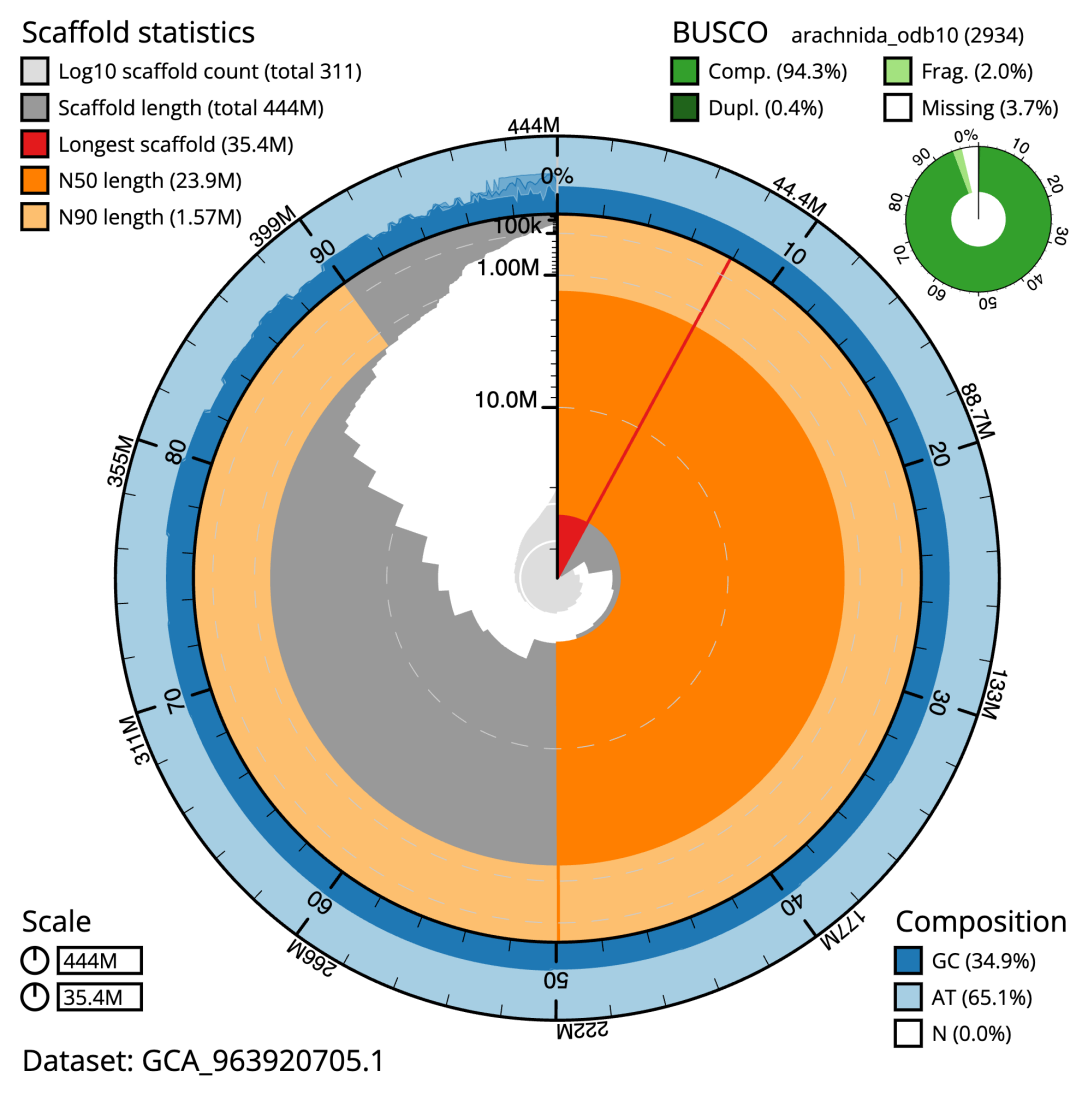
Genome assembly of *Odiellus spinosus*, qqOdiSpin1.1: metrics. The BlobToolKit snail plot shows N50 metrics and BUSCO gene completeness. The main plot is divided into 1,000 size-ordered bins around the circumference with each bin representing 0.1% of the 443,687,896 bp assembly. The distribution of scaffold lengths is shown in dark grey with the plot radius scaled to the longest scaffold present in the assembly (35,433,943 bp, shown in red). Orange and pale-orange arcs show the N50 and N90 scaffold lengths (23,904,202 and 1,574,974 bp), respectively. The pale grey spiral shows the cumulative scaffold count on a log scale with white scale lines showing successive orders of magnitude. The blue and pale-blue area around the outside of the plot shows the distribution of GC, AT and N percentages in the same bins as the inner plot. A summary of complete, fragmented, duplicated and missing BUSCO genes in the arachnida_odb10 set is shown in the top right. An interactive version of this figure is available at https://blobtoolkit.genomehubs.org/view/Odiellus_spinosus/dataset/GCA_963920705.1/snail.

**Figure 3.**
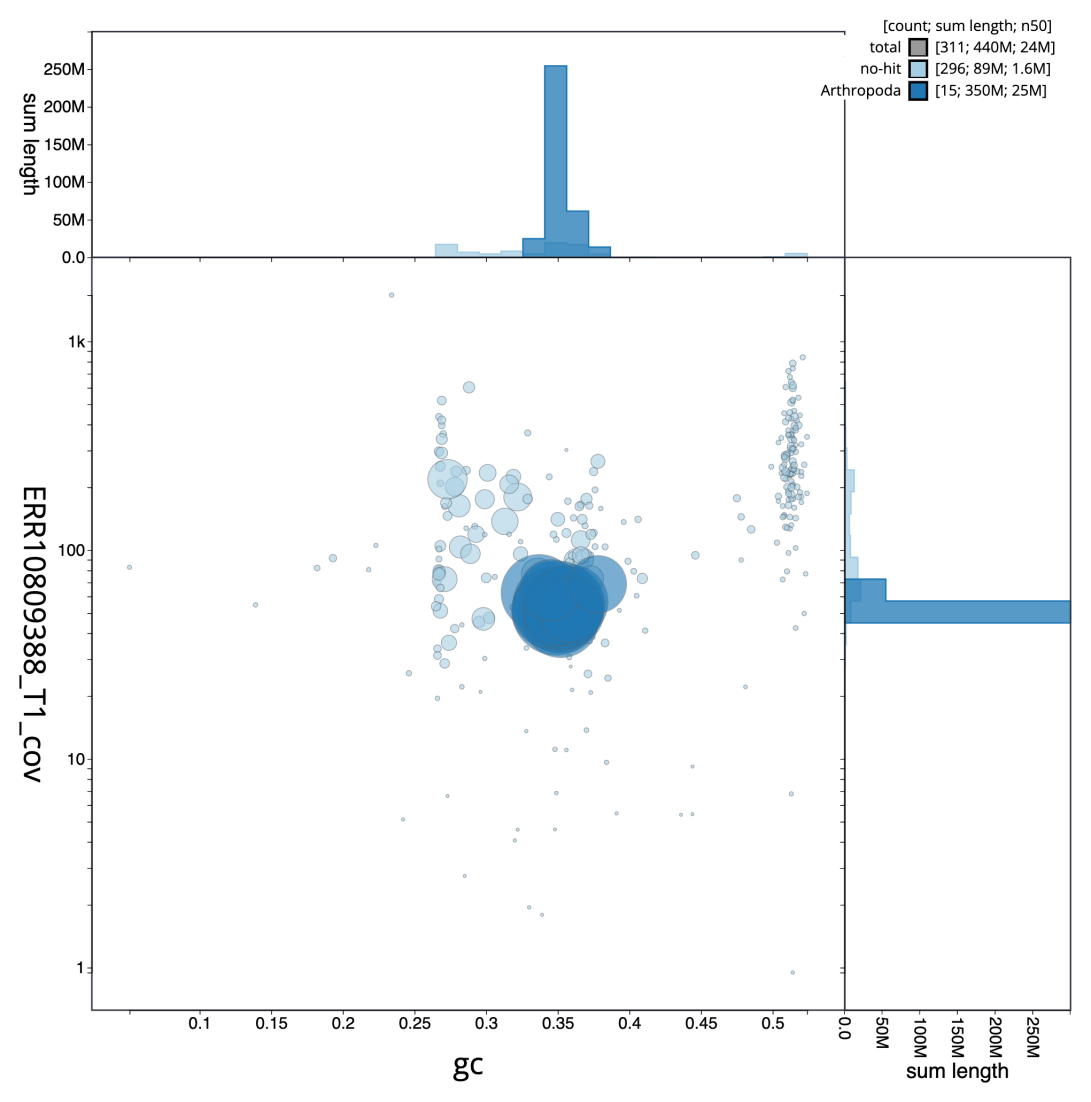
Genome assembly of *Odiellus spinosus*, qqOdiSpin1.1: BlobToolKit GC-coverage plot. Sequences are coloured by phylum. Circles are sized in proportion to sequence length. Histograms show the distribution of sequence length sum along each axis. An interactive version of this figure is available at https://blobtoolkit.genomehubs.org/view/Odiellus_spinosus/dataset/GCA_963920705.1/blob.

**Figure 4.**
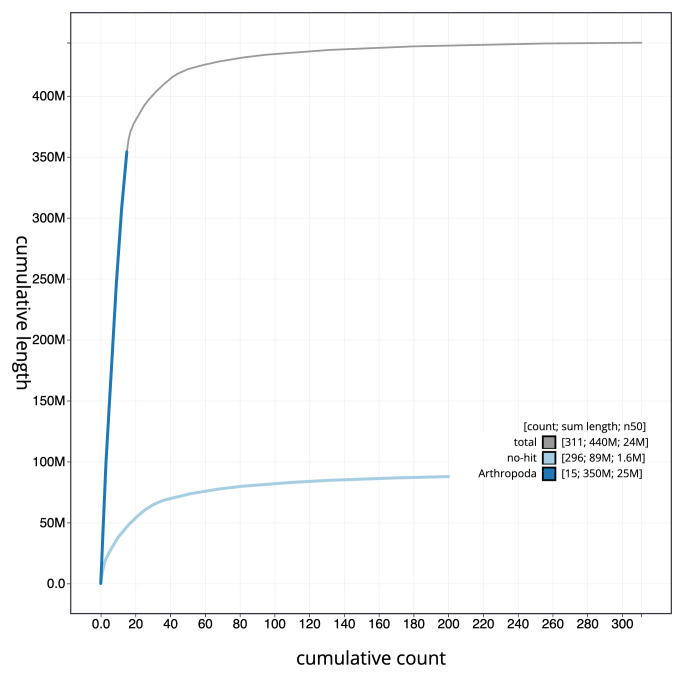
Genome assembly of *Odiellus spinosus* qqOdiSpin1.1: BlobToolKit cumulative sequence plot. The grey line shows cumulative length for all sequences. Coloured lines show cumulative lengths of sequences assigned to each phylum using the buscogenes taxrule. An interactive version of this figure is available at https://blobtoolkit.genomehubs.org/view/Odiellus_spinosus/dataset/GCA_963920705.1/cumulative.

**Figure 5.**
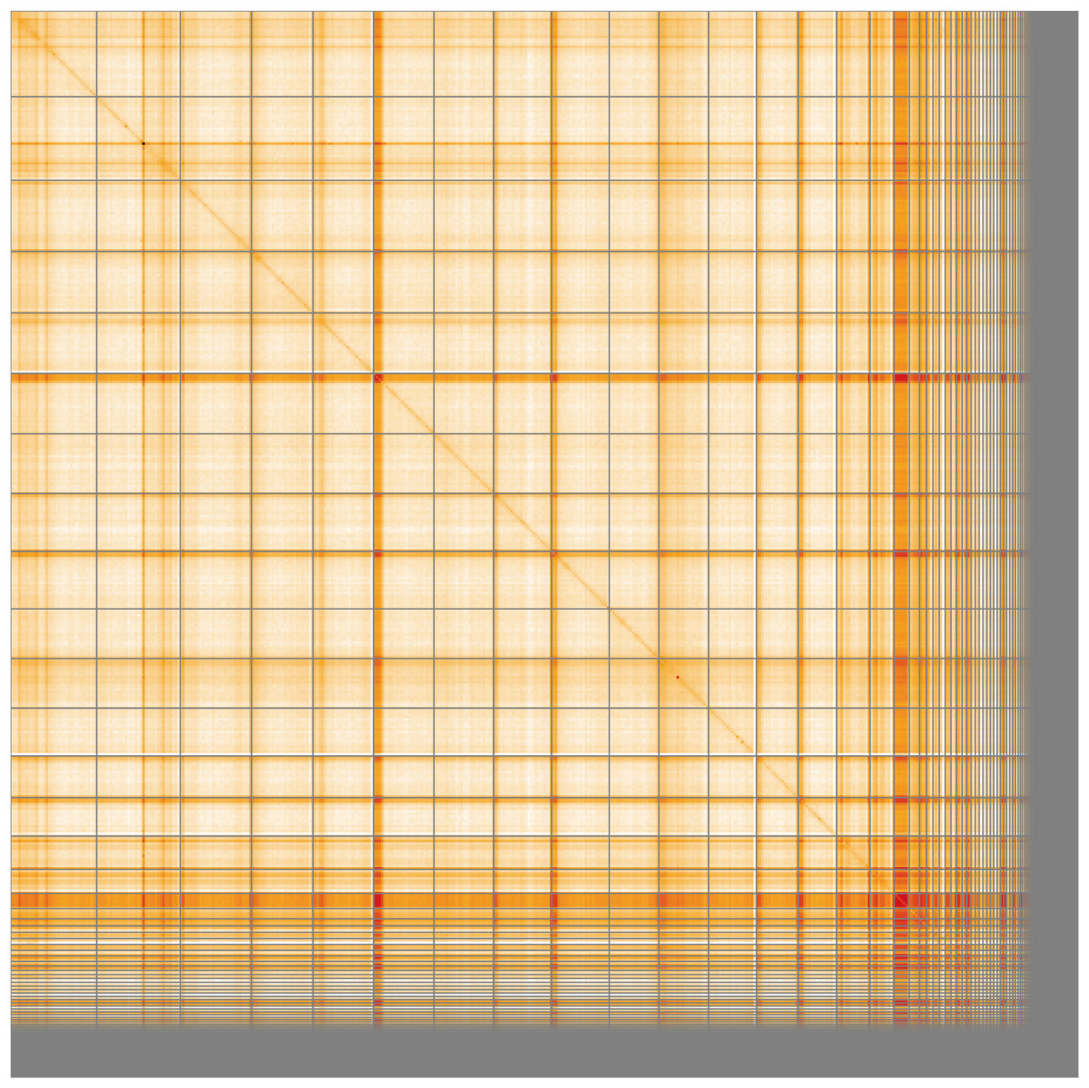
Genome assembly of *Odiellus spinosus* qqOdiSpin1.1: Hi-C contact map of the qqOdiSpin1.1 assembly, visualised using HiGlass. Chromosomes are shown in order of size from left to right and top to bottom. An interactive version of this figure may be viewed at https://genome-note-higlass.tol.sanger.ac.uk/l/?d=VyJk-NnhRWS8_9R5LO1qgg.

Manual assembly curation corrected 60 missing joins or mis-joins and 5 haplotypic duplications, reducing the scaffold number by 4.31%, and increasing the scaffold N50 by 10.52%. The final assembly has a total length of 443.70 Mb in 310 sequence scaffolds with a scaffold N50 of 23.9 Mb ( Table 2). The total count of gaps in the scaffolds is 230, with an average length of 200.00 bp. The snail plot in Figure 2 provides a summary of the assembly statistics, while the distribution of assembly scaffolds on GC proportion and coverage is shown in Figure 3. The cumulative assembly plot in Figure 4 shows curves for subsets of scaffolds assigned to different phyla. Most (82.91%) of the assembly sequence was assigned to 16 chromosomal-level scaffolds. Chromosome-scale scaffolds confirmed by the Hi-C data are named in order of size ( Figure 5; Table 3). While not fully phased, the assembly deposited is of one haplotype. Contigs corresponding to the second haplotype have also been deposited. The mitochondrial genome was also assembled and can be found as a contig within the multifasta file of the genome submission.

**Table 2.** Genome assembly data for *Odiellus spinosus*, qqOdiSpin1.1.

Genome assembly		
Assembly name	qqOdiSpin1.1	
Assembly accession	GCA_963920705.1	
*Accession of alternate haplotype*	*GCA_963920695.1*	
Span (Mb)	443.70	
Number of contigs	541	
Contig N50 length (Mb)	3.6	
Number of scaffolds	310	
Scaffold N50 length (Mb)	23.9	
Longest scaffold (Mb)	35.43	
Assembly metrics *		*Benchmark*
Consensus quality (QV)	60.6	*≥ 50*
*k*-mer completeness	100.0%	*≥ 95%*
BUSCO **	C:94.8%[S:94.4%,D:0.4%],F:1.6%,M:3.6%,n:2,934	*C ≥ 95%*
Percentage of assembly mapped to chromosomes	82.91%	*≥ 95%*
Sex chromosomes	Not identified	*localised homologous pairs*
Organelles	Mitochondrial genome: 16.07 kb	*complete single alleles*

* Assembly metric benchmarks are adapted from column VGP-2020 of “Table 1: Proposed standards and metrics for defining genome assembly quality” from Rhie *et al.* (2021). ** BUSCO scores based on the arachnida_odb10 BUSCO set using version 5.4.3. C = complete [S = single copy, D = duplicated], F = fragmented, M = missing, n = number of orthologues in comparison. A full set of BUSCO scores is available at https://blobtoolkit.genomehubs.org/view/Odiellus_spinosus/dataset/GCA_963920705.1/busco.

**Table 3.** Chromosomal pseudomolecules in the genome assembly of *Odiellus spinosus*, qqOdiSpin1.

INSDC accession	Name	Length (Mb)	GC%
OY987225.1	1	35.43	35.0
OY987226.1	2	34.49	35.0
OY987227.1	3	29.37	35.0
OY987228.1	4	25.33	35.5
OY987229.1	5	24.93	35.0
OY987230.1	6	24.91	33.5
OY987231.1	7	24.62	35.0
OY987232.1	8	20.45	35.0
OY987233.1	9	23.9	34.5
OY987234.1	10	23.73	36.0
OY987235.1	11	20.48	36.0
OY987236.1	12	19.72	35.0
OY987237.1	13	17.21	36.0
OY987238.1	14	15.87	34.5
OY987239.1	15	13.74	38.0
OY987240.1	16	9.92	34.5
OY987241.1	MT	0.02	24.0

The estimated Quality Value (QV) of the final assembly is 60.6 with *k*-mer completeness of 100.0%, and the assembly has a BUSCO v5.4.3 completeness of 94.8% (single = 94.4%, duplicated = 0.4%), using the arachnida_odb10 reference set ( *n* = 2,934).

Metadata for specimens, BOLD barcode results, spectra estimates, sequencing runs, contaminants and pre-curation assembly statistics are given at https://links.tol.sanger.ac.uk/species/2803876.

## Methods

### Sample acquisition

Adult specimens of *Odiellus spinosus* was collected from Feltham, England, UK (latitude 51.44, longitude –0.39) on 2020-10-01. The specimens were collected and identified by Sergio Henriques (Indianapolis Zoological Society) and preserved in liquid nitrogen. The specimen used for genome sequencing was specimen NHMUK014447001 (ToLID qqOdiSpin1), specimen NHMUK014447000 (ToLID qqOdiSpin2) was used for Hi-C sequencing, and specimen NHMUK014446999 (ToLID qqOdiSpin3) was used for RNA sequencing.

The initial identification was verified by an additional DNA barcoding process according to the framework developed by Twyford *et al*. (2024). A small sample was dissected from the specimens and stored in ethanol, while the remaining parts of the specimen were shipped on dry ice to the Wellcome Sanger Institute (WSI). The tissue was lysed, the COI marker region was amplified by PCR, and amplicons were sequenced and compared to the BOLD database, confirming the species identification ( Crowley *et al.*, 2023). Following whole genome sequence generation, the relevant DNA barcode region is also used alongside the initial barcoding data for sample tracking at the WSI ( Twyford *et al.*, 2024). The standard operating procedures for Darwin Tree of Life barcoding have been deposited on protocols.io ( Beasley *et al.*, 2023).

### Nucleic acid extraction

The workflow for high molecular weight (HMW) DNA extraction at the WSI Tree of Life Core Laboratory includes a sequence of core procedures: sample preparation; sample homogenisation, DNA extraction, fragmentation, and clean-up. In sample preparation, the qqOdiSpin1 sample was weighed and dissected on dry ice ( Jay *et al.*, 2023).

Tissue from the cephalothorax was homogenised using a PowerMasher II tissue disruptor ( Denton *et al.*, 2023a). HMW DNA was extracted using the Automated MagAttract v1 protocol ( Sheerin *et al.*, 2023). DNA was sheared into an average fragment size of 12–20 kb in a Megaruptor 3 system with speed setting 30 ( Todorovic *et al.*, 2023). Sheared DNA was purified by solid-phase reversible immobilisation ( Strickland *et al.*, 2023): in brief, the method employs a 1.8X ratio of AMPure PB beads to sample to eliminate shorter fragments and concentrate the DNA. The concentration of the sheared and purified DNA was assessed using a Nanodrop spectrophotometer and Qubit Fluorometer using the Qubit dsDNA High Sensitivity Assay kit. Fragment size distribution was evaluated by running the sample on the FemtoPulse system.

RNA was extracted from abdomen tissue of qqOdiSpin3 in the Tree of Life Laboratory at the WSI using the RNA Extraction: Automated MagMax™ *mir*Vana protocol ( do Amaral *et al.*, 2023). The RNA concentration was assessed using a Nanodrop spectrophotometer and a Qubit Fluorometer using the Qubit RNA Broad-Range Assay kit. Analysis of the integrity of the RNA was done using the Agilent RNA 6000 Pico Kit and Eukaryotic Total RNA assay.

Protocols developed by the WSI Tree of Life laboratory are publicly available on protocols.io ( Denton *et al.*, 2023b).

### Sequencing

Pacific Biosciences HiFi circular consensus DNA sequencing libraries were constructed according to the manufacturers’ instructions. Poly(A) RNA-Seq libraries were constructed using the NEB Ultra II RNA Library Prep kit. DNA and RNA sequencing was performed by the Scientific Operations core at the WSI on Pacific Biosciences Sequel IIe (HiFi) and Illumina NovaSeq 6000 (RNA-Seq) instruments. Hi-C data were also generated from cephalothorax tissue of qqOdiSpin2 using the Arima-HiC v2 kit. The Hi-C sequencing was performed using paired-end sequencing with a read length of 150 bp on the Illumina NovaSeq 6000 instrument.

### Genome assembly, curation and evaluation

***Assembly***

HiFi reads were assembled using the ‘sanger-tol/genomeassembly’ pipeline ( Krasheninnikova *et al.*, 2024). Original assembly of HiFi reads is performed using Hifiasm ( Cheng *et al.*, 2021) with the --primary option. Haplotypic duplications were identified and removed with purge_dups ( Guan *et al.*, 2020). Hi-C reads are further mapped with bwa-mem2 ( Vasimuddin *et al.*, 2019) to the primary contigs, which are further scaffolded using the provided Hi-C data ( Rao *et al.*, 2014) in YaHS ( Zhou *et al.*, 2023) using the --break option. Scaffolded assemblies are evaluated using Gfastats ( Formenti *et al.*, 2022), BUSCO ( Manni *et al.*, 2021) and MERQURY.FK ( Rhie *et al.*, 2020).

The mitochondrial genome was assembled using MitoHiFi ( Uliano-Silva *et al.*, 2023), which runs MitoFinder ( Allio *et al.*, 2020) and uses these annotations to select the final mitochondrial contig and to ensure the general quality of the sequence.

***Assembly curation***

The assembly was decontaminated using the Assembly Screen for Cobionts and Contaminants (ASCC) pipeline (article in preparation). Flat files and maps used in curation were generated in TreeVal ( Pointon *et al.*, 2023). Manual curation was primarily conducted using PretextView ( Harry, 2022), with additional insights provided by JBrowse2 ( Diesh *et al.*, 2023) and HiGlass ( Kerpedjiev *et al.*, 2018). Scaffolds were visually inspected and corrected as described by Howe *et al*. (2021). Any identified contamination, missed joins, and mis-joins were corrected, and duplicate sequences were tagged and removed. The process is documented at https://gitlab.com/wtsi-grit/rapid-curation (article in preparation).

***Evaluation of the final assembly***

The final assembly was post-processed and evaluated with the three Nextflow ( Di Tommaso *et al.*, 2017) DSL2 pipelines “sanger-tol/readmapping” ( Surana *et al.*, 2023a), “sanger-tol/genomenote” ( Surana *et al.*, 2023b), and “sanger-tol/blobtoolkit” ( Muffato *et al.*, 2024). The pipeline sanger-tol/readmapping aligns the Hi-C reads with bwa-mem2 ( Vasimuddin *et al.*, 2019) and combines the alignment files with SAMtools ( Danecek *et al.*, 2021). The sanger-tol/genomenote pipeline transforms the Hi-C alignments into a contact map with BEDTools ( Quinlan & Hall, 2010) and the Cooler tool suite ( Abdennur & Mirny, 2020), which is then visualised with HiGlass ( Kerpedjiev *et al.*, 2018). It also provides statistics about the assembly with the NCBI datasets ( Sayers *et al.*, 2024) report, computes *k*-mer completeness and QV consensus quality values with FastK and MERQURY.FK, and a completeness assessment with BUSCO ( Manni *et al.*, 2021).

The sanger-tol/blobtoolkit pipeline is a Nextflow port of the previous Snakemake Blobtoolkit pipeline ( Challis *et al.*, 2020). It aligns the PacBio reads with SAMtools and minimap2 ( Li, 2018) and generates coverage tracks for regions of fixed size. In parallel, it queries the GoaT database ( Challis *et al.*, 2023) to identify all matching BUSCO lineages to run BUSCO ( Manni *et al.*, 2021). For the three domain-level BUSCO lineage, the pipeline aligns the BUSCO genes to the Uniprot Reference Proteomes database ( Bateman *et al.*, 2023) with DIAMOND ( Buchfink *et al.*, 2021) blastp. The genome is also split into chunks according to the density of the BUSCO genes from the closest taxonomically lineage, and each chunk is aligned to the Uniprot Reference Proteomes database with DIAMOND blastx. Genome sequences that have no hit are then chunked with seqtk and aligned to the NT database with blastn ( Altschul *et al.*, 1990). All those outputs are combined with the blobtools suite into a blobdir for visualisation.

The genome assembly and evaluation pipelines were developed using the nf-core tooling ( Ewels *et al.*, 2020), use MultiQC ( Ewels *et al.*, 2016), and make extensive use of the Conda package manager, the Bioconda initiative ( Grüning *et al.*, 2018), the Biocontainers infrastructure ( da Veiga Leprevost *et al.*, 2017), and the Docker ( Merkel, 2014) and Singularity ( Kurtzer *et al.*, 2017) containerisation solutions. Table 4 contains a list of relevant software tool versions and sources.

**Table 4.** Software tools: versions and sources.

Software tool	Version	Source
BEDTools	2.30.0	https://github.com/arq5x/bedtools2
BLAST	2.14.0	ftp://ftp.ncbi.nlm.nih.gov/blast/executables/blast+/
BlobToolKit	4.3.7	https://github.com/blobtoolkit/blobtoolkit
BUSCO	5.4.3 and 5.5.0	https://gitlab.com/ezlab/busco
bwa-mem2	2.2.1	https://github.com/bwa-mem2/bwa-mem2
Cooler	0.8.11	https://github.com/open2c/cooler
DIAMOND	2.1.8	https://github.com/bbuchfink/diamond
fasta_windows	0.2.4	https://github.com/tolkit/fasta_windows
FastK	427104ea91c78c3b8b8b49f1a7d6bbeaa869ba1c	https://github.com/thegenemyers/FASTK
Gfastats	1.3.6	https://github.com/vgl-hub/gfastats
GoaT CLI	0.2.5	https://github.com/genomehubs/goat-cli
Hifiasm	0.16.1-r375	https://github.com/chhylp123/hifiasm
HiGlass	44086069ee7d4d3f6f3f0012569789ec138f42b84aa44357826c0b6753eb28de	https://github.com/higlass/higlass
Merqury.FK	d00d98157618f4e8d1a9190026b19b471055b22e	https://github.com/thegenemyers/MERQURY.FK
MitoHiFi	2	https://github.com/marcelauliano/MitoHiFi
MultiQC	1.14, 1.17, and 1.18	https://github.com/MultiQC/MultiQC
NCBI Datasets	15.12.0	https://github.com/ncbi/datasets
Nextflow	23.04.0-5857	https://github.com/nextflow-io/nextflow
PretextView	0.2	https://github.com/sanger-tol/PretextView
purge_dups	1.2.3	https://github.com/dfguan/purge_dups
samtools	1.16.1, 1.17, and 1.18	https://github.com/samtools/samtools
sanger-tol/ascc	-	https://github.com/sanger-tol/ascc
sanger-tol/genomeassembly	0.10.0	https://github.com/sanger-tol/genomeassembly
sanger-tol/genomenote	1.1.1	https://github.com/sanger-tol/genomenote
sanger-tol/readmapping	1.2.1	https://github.com/sanger-tol/readmapping
Seqtk	1.3	https://github.com/lh3/seqtk
Singularity	3.9.0	https://github.com/sylabs/singularity
TreeVal	1.0.0	https://github.com/sanger-tol/treeval
YaHS	1.2a	https://github.com/c-zhou/yahs

Table 4 contains a list of relevant software tool versions and sources.

### Wellcome Sanger Institute – Legal and Governance

The materials that have contributed to this genome note have been supplied by a Darwin Tree of Life Partner. The submission of materials by a Darwin Tree of Life Partner is subject to the **‘Darwin Tree of Life Project Sampling Code of Practice’**, which can be found in full on the Darwin Tree of Life website here. By agreeing with and signing up to the Sampling Code of Practice, the Darwin Tree of Life Partner agrees they will meet the legal and ethical requirements and standards set out within this document in respect of all samples acquired for, and supplied to, the Darwin Tree of Life Project.

Further, the Wellcome Sanger Institute employs a process whereby due diligence is carried out proportionate to the nature of the materials themselves, and the circumstances under which they have been/are to be collected and provided for use. The purpose of this is to address and mitigate any potential legal and/or ethical implications of receipt and use of the materials as part of the research project, and to ensure that in doing so we align with best practice wherever possible. The overarching areas of consideration are:

• Ethical review of provenance and sourcing of the material

• Legality of collection, transfer and use (national and international)

Each transfer of samples is further undertaken according to a Research Collaboration Agreement or Material Transfer Agreement entered into by the Darwin Tree of Life Partner, Genome Research Limited (operating as the Wellcome Sanger Institute), and in some circumstances other Darwin Tree of Life collaborators.

## Data Availability

European Nucleotide Archive: *Odiellus spinosus*. Accession number PRJEB59133; https://identifiers.org/ena.embl/PRJEB59133 ( Wellcome Sanger Institute, 2023). The genome sequence is released openly for reuse. The *Odiellus spinosus* genome sequencing initiative is part of the Darwin Tree of Life (DToL) project. All raw sequence data and the assembly have been deposited in INSDC databases. The genome will be annotated using available RNA-Seq data and presented through the Ensembl pipeline at the European Bioinformatics Institute. Raw data and assembly accession identifiers are reported in Table 1 and Table 2.

## Author information

Members of the Natural History Museum Genome Acquisition Lab are listed here: https://doi.org/10.5281/zenodo.7139035.

Members of the Darwin Tree of Life Barcoding collective are listed here: https://doi.org/10.5281/zenodo.4893703.

Members of the Wellcome Sanger Institute Tree of Life Management, Samples and Laboratory team are listed here: https://doi.org/10.5281/zenodo.10066175.

Members of Wellcome Sanger Institute Scientific Operations: Sequencing Operations are listed here: https://doi.org/10.5281/zenodo.10043364.

Members of the Wellcome Sanger Institute Tree of Life Core Informatics team are listed here: https://doi.org/10.5281/zenodo.10066637.

Members of the Tree of Life Core Informatics collective are listed here: https://doi.org/10.5281/zenodo.5013541.

Members of the Darwin Tree of Life Consortium are listed here: https://doi.org/10.5281/zenodo.4783558.

## References

[ref-1] Abdennur N Mirny LA : Cooler: scalable storage for Hi-C data and other genomically labeled arrays. *Bioinformatics.* 2020;36(1):311–316. 10.1093/bioinformatics/btz540 31290943 PMC8205516

[ref-2] Allio R Schomaker-Bastos A Romiguier J : MitoFinder: efficient automated large-scale extraction of mitogenomic data in target enrichment phylogenomics. *Mol Ecol Resour.* 2020;20(4):892–905. 10.1111/1755-0998.13160 32243090 PMC7497042

[ref-3] Altschul SF Gish W Miller W : Basic local alignment search tool. *J Mol Biol.* 1990;215(3):403–410. 10.1016/S0022-2836(05)80360-2 2231712

[ref-4] Bateman A Martin MJ Orchard S : UniProt: the universal protein knowledgebase in 2023. *Nucleic Acids Res.* 2023;51(D1):D523–D531. 10.1093/nar/gkac1052 36408920 PMC9825514

[ref-5] Beasley J Uhl R Forrest LL : DNA barcoding SOPs for the Darwin Tree of Life project. *protocols.io.* 2023; [Accessed 25 June 2024]. 10.17504/protocols.io.261ged91jv47/v1

[ref-6] Bennett A : Factors regulating the life cycle in a series of opiliones (Arthropoda, Arachnida) and external features in the embryology of one species, *Phalangium opilio* L. University of Leicester (United Kingdom),1962. Reference Source

[ref-7] Bristowe WS : The distribution of harvestmen (Phalangida) in Great Britain and Ireland, with notes on their names, enemies and food. *J Anim Ecol.* 1949;18(1):100–114. 10.2307/1584

[ref-8] Buchfink B Reuter K Drost HG : Sensitive protein alignments at Tree-of-Life scale using DIAMOND. *Nat Methods.* 2021;18(4):366–368. 10.1038/s41592-021-01101-x 33828273 PMC8026399

[ref-9] Challis R Kumar S Sotero-Caio C : Genomes on a Tree (GoaT): a versatile, scalable search engine for genomic and sequencing project metadata across the eukaryotic Tree of Life [version 1; peer review: 2 approved]. *Wellcome Open Res.* 2023;8:24. 10.12688/wellcomeopenres.18658.1 36864925 PMC9971660

[ref-10] Challis R Richards E Rajan J : BlobToolKit – interactive quality assessment of genome assemblies. *G3 (Bethesda).* 2020;10(4):1361–1374. 10.1534/g3.119.400908 32071071 PMC7144090

[ref-11] Cheng H Concepcion GT Feng X : Haplotype-resolved *de novo* assembly using phased assembly graphs with hifiasm. *Nat Methods.* 2021;18(2):170–175. 10.1038/s41592-020-01056-5 33526886 PMC7961889

[ref-12] Crowley L Allen H Barnes I : A sampling strategy for genome sequencing the British terrestrial arthropod fauna [version 1; peer review: 2 approved]. *Wellcome Open Res.* 2023;8:123. 10.12688/wellcomeopenres.18925.1 37408610 PMC10318377

[ref-13] da Veiga Leprevost F Grüning BA Alves Aflitos S : BioContainers: an open-source and community-driven framework for software standardization. *Bioinformatics.* 2017;33(16):2580–2582. 10.1093/bioinformatics/btx192 28379341 PMC5870671

[ref-14] Danecek P Bonfield JK Liddle J : Twelve years of SAMtools and BCFtools. *Gigascience.* 2021;10(2): giab008. 10.1093/gigascience/giab008 33590861 PMC7931819

[ref-15] Davidson MB : The British harvestman (Opiliones) fauna: 50 years of biodiversity change, and an annotated checklist. *Arachnology.* 2019;18(3):213–222. 10.13156/arac.2019.18.3.213

[ref-16] Denton A Oatley G Cornwell C : Sanger Tree of Life sample homogenisation: PowerMash. *protocols.io.* 2023a. 10.17504/protocols.io.5qpvo3r19v4o/v1

[ref-17] Denton A Yatsenko H Jay J : Sanger tree of life wet laboratory protocol collection V.1. *protocols.io.* 2023b. 10.17504/protocols.io.8epv5xxy6g1b/v1

[ref-18] Di Tommaso P Chatzou M Floden EW : Nextflow enables reproducible computational workflows. *Nat Biotechnol.* 2017;35(4):316–319. 10.1038/nbt.3820 28398311

[ref-19] Diesh C Stevens GJ Xie P : JBrowse 2: a modular genome browser with views of synteny and structural variation. *Genome Biol.* 2023;24(1): 74. 10.1186/s13059-023-02914-z 37069644 PMC10108523

[ref-20] do Amaral RJV Bates A Denton A : Sanger Tree of Life RNA extraction: automated MagMax ^TM^ mirVana. *protocols.io.* 2023. 10.17504/protocols.io.6qpvr36n3vmk/v1

[ref-22] Ewels P Magnusson M Lundin S : MultiQC: summarize analysis results for multiple tools and samples in a single report. *Bioinformatics.* 2016;32(19):3047–3048. 10.1093/bioinformatics/btw354 27312411 PMC5039924

[ref-21] Ewels PA Peltzer A Fillinger S : The nf-core framework for community-curated bioinformatics pipelines. *Nat Biotechnol.* 2020;38(3):276–278. 10.1038/s41587-020-0439-x 32055031

[ref-23] Formenti G Abueg L Brajuka A : Gfastats: conversion, evaluation and manipulation of genome sequences using assembly graphs. *Bioinformatics.* 2022;38(17):4214–4216. 10.1093/bioinformatics/btac460 35799367 PMC9438950

[ref-24] Grüning B Dale R Sjödin A : Bioconda: sustainable and comprehensive software distribution for the life sciences. *Nat Methods.* 2018;15(7):475–476. 10.1038/s41592-018-0046-7 29967506 PMC11070151

[ref-25] Guan D McCarthy SA Wood J : Identifying and removing haplotypic duplication in primary genome assemblies. *Bioinformatics.* 2020;36(9):2896–2898. 10.1093/bioinformatics/btaa025 31971576 PMC7203741

[ref-26] Harry E : PretextView (Paired REad TEXTure Viewer): A desktop application for viewing pretext contact maps.2022; [Accessed 19 October 2022]. Reference Source

[ref-27] Hillyard PD Sankey JH : Harvestmen: keys and notes for the identification of the species. *Brill Archive.* 1989;4 Reference Source

[ref-28] Howe K Chow W Collins J : Significantly improving the quality of genome assemblies through curation. *GigaScience.* Oxford University Press.2021;10(1): giaa153. 10.1093/gigascience/giaa153 33420778 PMC7794651

[ref-29] Jay J Yatsenko H Narváez-Gómez JP : Sanger Tree of Life sample preparation: triage and dissection. *protocols.io.* 2023. 10.17504/protocols.io.x54v9prmqg3e/v1

[ref-30] Kerpedjiev P Abdennur N Lekschas F : HiGlass: web-based visual exploration and analysis of genome interaction maps. *Genome Biol.* 2018;19(1): 125. 10.1186/s13059-018-1486-1 30143029 PMC6109259

[ref-31] Krasheninnikova K Qi G Muffato M : sanger-tol/genomeassembly: v0.10.0 - Hideous Zippleback. *Zenodo.* 2024. Reference Source

[ref-32] Kurtzer GM Sochat V Bauer MW : Singularity: scientific containers for mobility of compute. *PLoS One.* 2017;12(5): e0177459. 10.1371/journal.pone.0177459 28494014 PMC5426675

[ref-33] Li H : Minimap2: pairwise alignment for nucleotide sequences. *Bioinformatics.* 2018;34(18):3094–3100. 10.1093/bioinformatics/bty191 29750242 PMC6137996

[ref-34] Manni M Berkeley MR Seppey M : BUSCO update: novel and streamlined workflows along with broader and deeper phylogenetic coverage for scoring of eukaryotic, prokaryotic, and viral genomes. *Mol Biol Evol.* 2021;38(10):4647–4654. 10.1093/molbev/msab199 34320186 PMC8476166

[ref-35] Merkel D : Docker: lightweight Linux containers for consistent development and deployment. *Linux.* 2014;2014(239): 2. Reference Source

[ref-36] Muffato M Butt Z Challis R : sanger-tol/blobtoolkit: v0.3.0 – Poliwag.2024. 10.5281/zenodo.10649272

[ref-37] Pickard-Cambridge O : Monograph of the British Phalangidea or harvestmen. *Proceedings of the Dorset Natural History and Antiquarian Field Club, Sherborne/Dorchester.* 1890;11:163–216.

[ref-38] Pointon DL Eagles W Sims Y : sanger-tol/treeval v1.0.0 – Ancient Atlantis.2023. 10.5281/zenodo.10047654

[ref-39] Quinlan AR Hall IM : BEDTools: a flexible suite of utilities for comparing genomic features. *Bioinformatics.* 2010;26(6):841–842. 10.1093/bioinformatics/btq033 20110278 PMC2832824

[ref-40] Rao SSP Huntley MH Durand NC : A 3D map of the human genome at kilobase resolution reveals principles of chromatin looping. *Cell.* 2014;159(7):1665–1680. 10.1016/j.cell.2014.11.021 25497547 PMC5635824

[ref-41] Rhie A McCarthy SA Fedrigo O : Towards complete and error-free genome assemblies of all vertebrate species. *Nature.* 2021;592(7856):737–746. 10.1038/s41586-021-03451-0 33911273 PMC8081667

[ref-42] Rhie A Walenz BP Koren S : Merqury: reference-free quality, completeness, and phasing assessment for genome assemblies. *Genome Biol.* 2020;21(1): 245. 10.1186/s13059-020-02134-9 32928274 PMC7488777

[ref-43] Rozwałka R Rutkowski T Sienkiewicz P : New data on the occurence of two invasive harvestmen species - *Odiellus spinosus* (Bosc) and *Lacinius dentiger* (C. L. Koch) in Poland). *Fragmenta Faunistica.* 2013;56(1):47–54. 10.3161/00159301FF2013.56.1.047

[ref-44] Sayers EW Cavanaugh M Clark K : GenBank 2024 update. *Nucleic Acids Res.* 2024;52(D1):D134–D137. 10.1093/nar/gkad903 37889039 PMC10767886

[ref-45] Shah M Coulson S : Artportalen (Swedish Species Observation System). Version 92.256. SLU Artdatabanken. Occurrence dataset.2021; [Accessed 17 June 2024]. 10.15468/kllkyl

[ref-46] Sheerin E Sampaio F Oatley G : Sanger Tree of Life HMW DNA extraction: automated MagAttract v.1. *protocols.io.* 2023; [Accessed 21 November 2023]. 10.17504/protocols.io.x54v9p2z1g3e/v1

[ref-47] Spoek GL : The Opilionida (Arachnida) of the Netherlands. *Zoologische Verhandelingen.* 1963;63(1):1–70. Reference Source

[ref-48] Strickland M Cornwell C Howard C : Sanger Tree of Life fragmented DNA clean up: manual SPRI. *protocols.io.* 2023. 10.17504/protocols.io.kxygx3y1dg8j/v1

[ref-49] Surana P Muffato M Qi G : sanger-tol/readmapping: sanger-tol/readmapping v1.1.0 - Hebridean Black (1.1.0). *Zenodo.* 2023a. 10.5281/zenodo.7755669

[ref-50] Surana P Muffato M Sadasivan Baby C : sanger-tol/genomenote (v1.0.dev). *Zenodo.* 2023b. 10.5281/zenodo.6785935

[ref-51] Todorovic M Sampaio F Howard C : Sanger Tree of Life HMW DNA fragmentation: diagenode Megaruptor®3 for PacBio HiFi. *protocols.io.* 2023. 10.17504/protocols.io.8epv5x2zjg1b/v1

[ref-52] Toft S : Ten years after the invasion: *Dicranopalpus ramosus* and *Odiellus spinosus* (Opiliones, Phalangiidae) in Denmark. *Arachnologische Mitteilungen: Arachnology Letters.* 2018;56(1):1. 10.30963/aramit5601

[ref-53] Twyford AD Beasley J Barnes I : A DNA barcoding framework for taxonomic verification in the Darwin Tree of Life Project [version 1; peer review: awaiting peer review]. *Wellcome Open Res.* 2024;9:339. 10.12688/wellcomeopenres.21143.1 39386966 PMC11462125

[ref-54] Uliano-Silva M Ferreira JGRN Krasheninnikova K : MitoHiFi: a python pipeline for mitochondrial genome assembly from PacBio high fidelity reads. *BMC Bioinformatics.* 2023;24(1): 288. 10.1186/s12859-023-05385-y 37464285 PMC10354987

[ref-55] Van de Poel S Mestdagh C De Smedt P : Habitat, phenology and distribution of the expanding harvestman *Odiellus spinosus* in Belgium. *Journal of the Belgian Arachnological Society.* 2021;36(2):55–63. Reference Source

[ref-56] Vasimuddin M Misra S Li H : Efficient architecture-aware acceleration of bwa-mem for multicore systems.In: *2019 IEEE International Parallel and Distributed Processing Symposium (IPDPS).*IEEE,2019;314–324. 10.1109/IPDPS.2019.00041

[ref-59] Wellcome Sanger Institute: The genome sequence of the harvestman spider, *Odiellus spinosus* (Bosc, 1792).European Nucleotide Archive. [dataset]. accession number PRJEB59133.2023.

[ref-57] Wijnhoven H : De Nederlandse hooiwagens (Opiliones). *Entomologische Tabellen.* 2009;3:1–118. Reference Source

[ref-58] Zhou C McCarthy SA Durbin R : YaHS: yet another Hi-C Scaffolding tool. *Bioinformatics.* 2023;39(1): btac808. 10.1093/bioinformatics/btac808 36525368 PMC9848053

