# Supplementary material for: The genome sequence of the harvestman spider, Odiellus spinosus (Bosc, 1792)
Source: Wellcome Open Res. 2024 Jul 26;9:407. [Version 1] doi: 10.12688/wellcomeopenres.22760.1 (PMC11503004; doi:10.12688/wellcomeopenres.22760.1)
Supplement: Peer review history

# Reviewer report: Reviewer response for version 1

**DOI:** 10.21956/wellcomeopenres.25064.r104497

**Published:** 2024-10-24

**Authors:** Chetan Munegowda

**Affiliations:** 1 Justus Liebig University Giessen, Giessen, Hesse, Germany

The manuscript presents a high-quality genome assembly of a female *Odiellus spinosus*, generated as part of the Darwin Tree of Life Project. The procedures for sample collection, sequencing, and assembly are methodologically robust, and sufficient details are provided to enable replication of the study. Additionally, all relevant data have been made publicly accessible, ensuring transparency and facilitating further research.

 However, it would be beneficial to clarify that this genome assembly is not generated from a single individual, as suggested in the abstract, since the Hi-C sequencing data used for scaffolding were obtained from a different specimen. This clarification would enhance the accuracy and transparency of the manuscript.

Are sufficient details of methods and materials provided to allow replication by others?

Yes

Is the rationale for creating the dataset(s) clearly described?

Yes

Are the datasets clearly presented in a useable and accessible format?

Yes

Are the protocols appropriate and is the work technically sound?

Yes

Reviewer Expertise:

arachnid genomics, transcriptomics, and evo-devo

I confirm that I have read this submission and believe that I have an appropriate level of expertise to confirm that it is of an acceptable scientific standard.

# Reviewer report: Reviewer response for version 1

**DOI:** 10.21956/wellcomeopenres.25064.r96361

**Published:** 2024-09-11

**Authors:** Madeleine Aase-Remedios

**Affiliations:** 1 Durham University, Durham, England, UK

The authors present a high-quality genome assembly for the harvestman *Odiellus spinosus* for public use using state of the art methods. Rationale for this species is not explicitly given, but clearly it falls under the larger aims of the DToL project.

 The background is thorough and gives a good description of the biology and ecology of the species.

 The methods report the programs used consistent with the pipeline, but do not specify the details on parameters or manual curation that would make this fully reproducible, but the extent the methods are reported is up to standard.

 The annotation is already available on Ensembl Rapid Release which I appreciate as many other DToL genomes do not make the annotation available at the time the genome assembly is released. This massively increases the utility of this resource!

 There is a minor grammatical error in the first sentence of the Sample acquisition section of the Methods: it should read "Adult specimens of *Odiellus spinosus* were collected" (not was).

Are sufficient details of methods and materials provided to allow replication by others?

Partly

Is the rationale for creating the dataset(s) clearly described?

Yes

Are the datasets clearly presented in a useable and accessible format?

Yes

Are the protocols appropriate and is the work technically sound?

Yes

Reviewer Expertise:

Arachnid development, genomics, and transcriptomics

I confirm that I have read this submission and believe that I have an appropriate level of expertise to confirm that it is of an acceptable scientific standard.
